# Contactless Impedance Sensors and Their Application to Flow Measurements

**DOI:** 10.3390/s130302786

**Published:** 2013-02-27

**Authors:** František Opekar, Petr Tůma, Karel Štulík

**Affiliations:** 1 Department of Analytical Chemistry, Faculty of Science, Charles University in Prague, Albertov 2030, CZ-128 43 Prague 2, Czech Republic; E-Mails: opekar@natur.cuni.cz (F.O.); stulik@natur.cuni.cz (K.Š.); 2 Institute of Biochemistry, Third Faculty of Medicine, Cell and Molecular Biology, Charles University in Prague, Ruská 87, CZ-100 00 Prague 10, Czech Republic

**Keywords:** contactless detector, conductivity detection, impedance detector, permittivity detection

## Abstract

The paper provides a critical discussion of the present state of the theory of high-frequency impedance sensors (now mostly called contactless impedance or conductivity sensors), the principal approaches employed in designing impedance flow-through cells and their operational parameters. In addition to characterization of traditional types of impedance sensors, the article is concerned with the use of less common sensors, such as cells with wire electrodes or planar cells. There is a detailed discussion of the effect of the individual operational parameters (width and shape of the electrodes, detection gap, frequency and amplitude of the input signal) on the response of the detector. The most important problems to be resolved in coupling these devices with flow-through measurements in the liquid phase are also discussed. Examples are given of cell designs for continuous flow and flow-injection analyses and of detection systems for miniaturized liquid chromatography and capillary electrophoresis. New directions for the use of these sensors in molecular biology and chemical reactors and some directions for future development are outlined.

## 1. Introduction

The rapidly growing demands on sensing, detection, and determination of a vast variety of compounds in wide ranges of test systems under very different conditions emphasize the need for intense methodological research. To meet these requirements, all imaginable measuring principles are being tested and, where possible, developed and applied. A good example is the revitalization of high-frequency measurement of the electrical impedance of test systems. This principle is, in fact, rather old; for reviews see, e.g., references [1,2]. However, its analytical application was rare and mostly limited to high-frequency titrations and to some determinations of water in non-aqueous samples. Renewed interest in these measurements emerged quite recently (during the nineteen nineties), primarily for several reasons:

(a) Technical developments have made measurements simple, flexible and cheap;
(b) The use of substantially lower frequencies of the applied signal has led to better definition of the output, which is more easily interpreted;
(c) Rapidly developing high-performance separations in the liquid phase, especially those on a microscale or on chips, require appropriate detection techniques;
(d) The universal detection character and the separation of the sensing elements from the test medium make the technique attractive for analyses of complex mixtures of similar analytes.

High-frequency impedance detection, now mostly called “capacitively coupled contactless conductivity detection”, abbreviated as C^4^D, is mostly employed for detection in capillary zone electrophoresis (CE); reviews of recent developments in this field can be found, e.g., in references [3–8]. The present paper deals with less traditional geometric arrangements of the detection cells and applications of contactless impedance detectors. The results obtained primarily in the authors' laboratories are critically discussed and assessed in the context of global research. Some tentative directions for the future are also outlined.

## 2. Basic Theoretical Considerations

**Figure 1.**
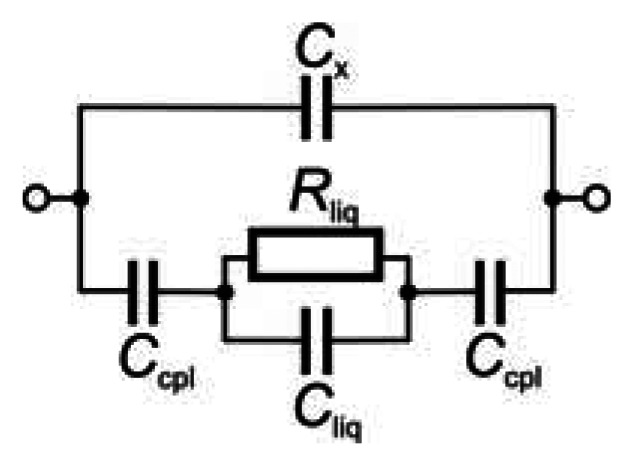
A simplified scheme of the equivalent electric circuit for the contactless impedance cell with connections to the input high-frequency voltage source and the output signal meter. For discussion and symbols explanation see the text.

A simplified electric equivalent circuit of a contactless impedance cell, permitting sufficiently detailed theoretical description of the cell operation, is depicted in Figure 1. A high-frequency alternating (usually sine-wave) voltage is applied to one electrode and the interaction of the alternating electrical field with the test medium is monitored at the other electrode, usually in the form of an alternating current signal. The two electrodes are separated from the test medium (usually liquid) by a dielectric layer and represent two capacitors characterized by coupling capacitance *C*_cpl_; *C*_cpl_ depends predominantly on the thickness and permittivity of the dielectric layer. The electrical behaviour of the test medium appears as a parallel combination of the liquid resistance (*R*_liq_) and the liquid capacitance (*C*_liq_). Part of the electrical energy applied always strays from the test medium, passes along the surface of the dielectric or through its interior and appears as stray (parasitic) capacitance *C*_x_, parallel to the main passageway. The parasitic effect of the stray capacitance is sometimes eliminated by placing a shielding foil between the electrodes [9,10].

The analytical signal is given by the cell impedance, *Z*, defined by the familiar general equation:

(1) Z=R+iX

where the real term, resistance *R*, is a function of the cell geometry and of the electrical conductivity of the test medium. The imaginary term, capacitance *X*, also depends on the geometric parameters and further on the relative permittivities of the test medium and of the dielectric, and on the angular frequency, *ω*, or ordinary frequency, *f*, of the input alternating signal (*ω* = 2π*f*); *i* is the imaginary unit.

It can be seen that the cell behavior depends on a number of experimental parameters and it should be emphasized that all these parameters affect one another, so that they must be considered together when studying the behavior of a particular cell under particular conditions. It is also evident that the set of experimental conditions determines whether the resistance term of Equation (1) predominates—this is the case of the contactless conductivity detection which is mostly used at present, or the capacitance is more important (dielectrometry).

The impedance of the electric equivalent circuit in Figure 1 can be calculated from Equation (2):

(2) $$Z=Z_{1}Z_{2}/(Z_{1}+Z_{2})$$

where *Z*_1_ is the impedance of the bottom branch of equivalent circuit, which equals to:

(3) $$Z_{1}=\frac{R_{\text{liq}}\cdot \left(-i/\omega C_{\text{liq}}\right)}{R_{\text{liq}}-i/\omega C_{\text{liq}}}-2i/\omega C_{\text{cpl}}$$

If *R*_liq_ ≪ (−*i*/*ωC*_liq_), the effect of the solution capacitance can be neglected, Equation (3) is simplified to *Z*_1_ = *R_liq_* − *2i*/*ωC_cpl_* and the sensor works primarily as a conductivity detector. If *R*_liq_ ≫ (−*i*/*ωC*_liq_), the effect of the solution resistivity can be neglected, Equation (3) is simplified to *Z*_1_ = −*i/C_liq_* − *2i*/*ωC_cpl_* and the sensor works primarily as a dielectrometric detector.

*Z*_2_ is the impedance determined by stray capacitance *C*_x_ (upper branch of equivalent circuit):

(4) $$Z_{2}=-i/\omega C_{x}$$

The analytical signal is given by the difference, Δ*Z*, between the impedance obtained in the presence of the analyte within the cell, *Z_A_*, and that of the cell containing only a carrier medium, *Z_E_*:

(5) $$\Delta Z=Z_{A}-Z_{E}$$

The measured quantity is usually the alternating current:

(6) I=U/|ΔZ|

where *U* is the amplitude of the input alternating voltage.

## 3. Design of Impedance Cells

**Figure 2.**
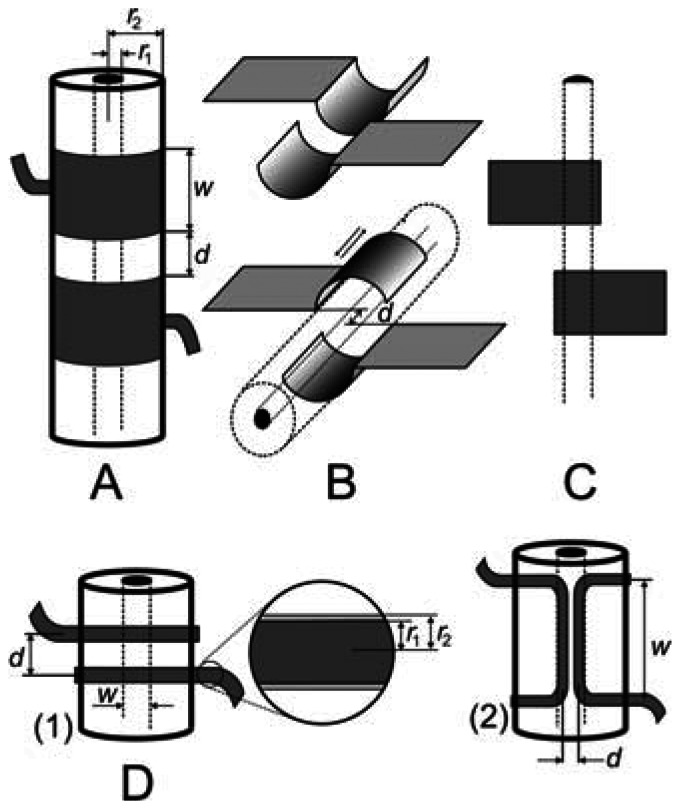
Examples of contactless impedance cell designs used mostly for conductometric detection. (**A**) tubular electrodes; (**B**) semitubular electrodes placed either in series or opposite one other; (**C**) planar electrodes; (**D**) insulated wire electrodes oriented across the tube (**1**) or placed axially inside the tube (**2**). *w*—geometric length of the electrode; *d*—width of the gap between the electrodes; *r*_1_ and *r*_2_—the inner and outer radii of the tube (capillary) with the test solution or the radii of the bare wire and the wire with the insulating film.

**Figure 3.**
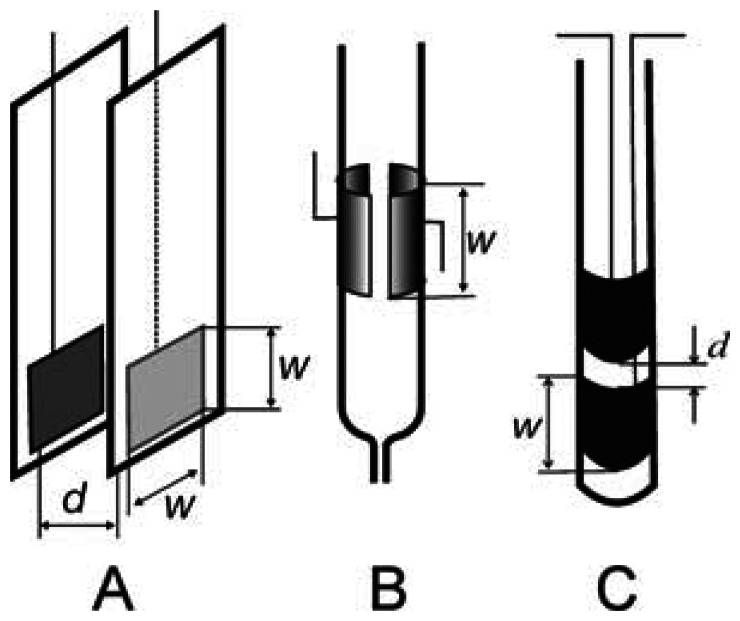
Examples of contactless impedance cell designs used mostly for dielectrometric detection. (**A**) planar electrodes oriented opposite one other; (**B**) flow-through cell with semitubular electrodes on the outside tube wall; (**C**) dipping cell with cylindrical electrodes placed one above the other.

Typical examples of the cell geometries discussed in this paper can be seen in Figures 2 and 3; the test solution either flows through the cell (Figures 2 and 3B) or the cell is immersed in the solution (Figure 3A,C). Tubular systems (Figure 2A) are common in flow-through applications, primarily liquid chromatography and capillary electrophoresis. Semitubular electrodes (Figure 2B) can also be used with advantage in these methods (see Section 4.1). Planar geometries (Figure 2C) are useful in microfluidic systems, e.g., chip electrophoresis, or lab-on-the-chip systems. Further geometric arrangements have also been studied, e.g., a pair of thin insulated wires placed inside tubing containing the test solution (Figure 2D).

The cell geometric arrangements mentioned above are primarily employed in conductometric detection. The cells in Figure 3 are predominantly used for dielectrometric detection: a pair of insulated planar electrodes placed opposite one another at a short distance (Figure 3A), tubular flow-through cell with electrodes placed on the outside wall of the tube (Figure 3B) or cylindrical dipping cell with electrodes protected from direct contact with the test environment by plastic foil (Figure 3C). It should be added that the separation of the detection cells into conductometric a dielectrometric is only illustrative. The conductivity or dielectrometric behavior of the detector depends on the geometry of the cell employed and also on a number of other parameters, such as the thickness and permittivity of the dielectric employed, the specific conductivity and the permittivity of the measured solution and the frequency of the input signal.

The detector electronics used is mostly based on the measuring principle described in one of the first papers at the beginning of the renaissance of contactless conductivity detection in capillary electrophoresis [11]. An alternating voltage produced by a function generator is fed to one of the detection cell electrodes and the electric current passing through the cell is monitored at the other electrode, using a current-voltage converter. The analytical signal—a voltage dependent on the cell impedance—is displayed after processing and amplification. The electronic circuitry is mostly assembled using operational amplifiers. The electronic circuit has recently been improved using modern electronic components [12].

The other approach to the monitoring of the cell signal was described in reference [13]. An un-modulated or an amplitude-modulated alternating voltage was applied to the tubular cell, and the AC current passing through the cell was treated by a TDA 1072A integrated circuit originally designed for application to medium-wave AM (amplitude modulated) radio receivers. The obtained signal depended on the conductivity of the solution inside the cell. Under optimum conditions, the solution conductance was measured in a range from *ca.* 10 to 700 μs cm^−1^. This detector was used to measure the conductivities of various drinking waters and the values obtained were in a good agreement with those obtained from a commercial contact conductometer.

Tubular geometries (Figure 2A) have so far been studied most thoroughly; the *R_liq_* and *C_cpl_* values in Equation (3) can be expressed as:

(7) $$R_{\text{liq}}=\frac{1}{\kappa }\cdot \frac{l}{\pi r_{1}^{2}}$$

and:

(8) $$C_{\text{cpl}}=\frac{2\pi {ɛ}_{0}{ɛ}_{r}w}{\ln(r_{2}/r_{1})}$$

where *l* is the length of the detection cell, which is primarily given by the gap between electrodes (*d*) and also a certain part of the electrode length, *w*_eff_, contributes to the detection cell length (see Section 4.2); *κ* is the specific conductance and *ɛ*_0_ and *ɛ*_r_ are the permittivities of a vacuum and the dielectric used, respectively; the other symbols are defined in the caption for Figure 2. The resistance, *R_liq_*, [Equation (7)] is calculated for a solution with specific conductivity *κ*, inside a cylindrical detection cell with length *l* and radius *r*_1_; *C_cpl_* [Equation (8)] is calculated as the capacitance of a cylindrical detection cell with external and internal radii of *r*_2_ and *r*_1_ an length of *w*, whose walls form a dielectric with relative permittivity *ɛ*_r_. Employing the theoretical basis described, the behavior of the cells can be modeled for various sets of experimental conditions. The stray capacitance must be found empirically; its values are usually of the order of hundredths to units of pF. The modeled and experimental dependences are presented in normalized forms, to enable combined plotting and comparison. The absolute values of the model and experimental quantities are usually rather different, due to the considerable simplification of the model, but the shapes of the dependences match quite well and permit prediction and optimization of the performance of the system.

## 4. Operational Parameters of Impedance Cells

### 4.1. The Effect of the Electrode Shape on the Signal Measured

As mentioned above, tubular cell geometries are mostly used for detection in flowing liquids. Our measurements [14,15] have demonstrated that the replacement of classical tubular electrodes (Figure 2A) by semitubular ones (Figure 2B) leads to negligible changes in the detector parameters and offers certain advantages. The metal foil semitubular electrodes were cemented in a groove made in a plexiglass plate. The groove dimension exactly matched the outer diameter of the analytical separation capillary, which was tightly pressed into the channel by an auxiliary plate. This arrangement enables ready replacement of the capillary if necessary. There is no air gap between the capillary surface and the electrodes that would contribute to the detector noise [10]. Tight coupling between the electrodes and the capillary surface can also be achieved in the tubular arrangement if the electrodes are made of conducting lacquer which is painted around the capillary [11,16]; in this case, the electrodes become a non-detachable part of the capillary.

It has also been shown that even planar electrodes can be used, with the separation capillary pressed onto them [15]. The design is so simple that the electrodes can be created directly on the printed board containing the detector electronics. The detector sensitivity is not decreased substantially and amounts to approximately one half that obtained with tubular electrodes. A thin aluminum foil can be used to advantage to prepare the electrodes for these detectors. These electrodes, similar to those prepared on the capillary surface using conductive lacquers, are very thin and thus the parasitic signal transfer between them outside the test solution is small. Therefore, there is no need to place a shielding foil between the electrodes, which is often necessary when using thicker tubular electrodes. Shielding foil minimizes the stray capacitance but does not eliminate it completely [10]. Moreover, it makes the cells more complicated and makes it difficult to implement simultaneous optical detection between the electrodes.

These aluminium foil electrodes have also been successfully used for detection in electrophoretic separations on a chip [17,18]. In this case, two planar electrodes are placed side by side on the outer wall of the chip lid, close to the output end of the separation channel (similar to the arrangement depicted in Figure 2C).

### 4.2. The Effect of the Electrode Width

**Figure 4.**
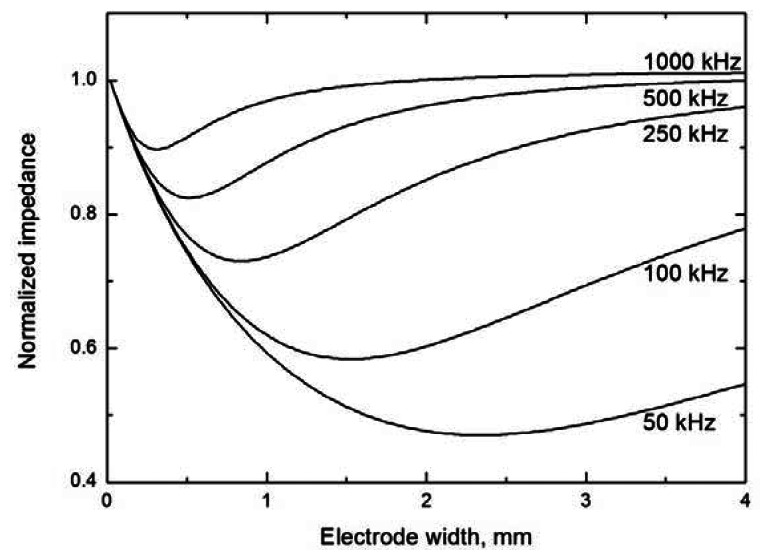
Model dependences of the impedance on the electrode width for various input voltage frequencies (specified next to the curves, in kHz); all the model calculations are based on the equivalent circuit in Figure 1. The modelling parameters are: silica capillary, *r*_1_ = 37.5 μm, *r*_2_ = 190 μm, *ε_r_* = 4.3; *d* = 1 mm and *C_x_* = 0.1 pF (estimated); the capillary is filled with 20 mM morpholinoethanesulfonic acid/histidine (MES/His) solution (pH 6.1), conductivity *κ* = 4.7 × 10^−2^ S m^−1^. Because of the large difference in the absolute values of the impedance, |*Z*|, the dependences are depicted in normalized form for the individual frequencies, |*Z*| = 1 for *w* → 0.

It has been found [16] that the width of tubular electrodes (Figure 2A) has a small effect on the magnitude of the measured signal. On the other hand, the optimum applied voltage frequency strongly depends on the electrode width and equals from about 400 to 600 kHz for narrow (2 mm) electrodes [11,19] and is substantially lower for wider ones, 20 to 40 kHz [16] or 40 to 100 kHz [20] for electrodes 15 to 50 mm wide, respectively. These observations should be explained by theoretical model based on the solution of the equivalent circuit (Figure 1) with the electrode width as a variable. The performed simulation (see Figure 4) indicates that the dependence of the cell impedance on the electrode width passes through a minimum and the current signal follows a trajectory characterized by the lowest impedance on passage through the cell. Consequently, the signal does not pass through the whole electrode width, *w*, but only through a small part called the effective width, *w*_eff_[21]. It can be seen in Figure 4 that the effective electrode width significantly varies with varying signal frequency, decreasing with increasing frequency. Its value is smaller than 1 mm for frequencies above 200 kHz and reaches only tenths of a millimetre at frequencies around 1 MHz. For this reason, the effective width is often neglected and the only gap is considered in expression of the cell resistance *R_liq_*. The overall length of the detection space equals 2 *w*_eff_ + *d* and determines the effective detector volume, which depends on the signal frequency. Cells with narrow electrodes can be used at high frequencies without decreasing the detection sensitivity; wide electrodes permit the use of lower frequencies. These conclusions are confirmed by the results published in reference [22].

### 4.3. The Effect of the Gap between the Electrodes

**Figure 5.**
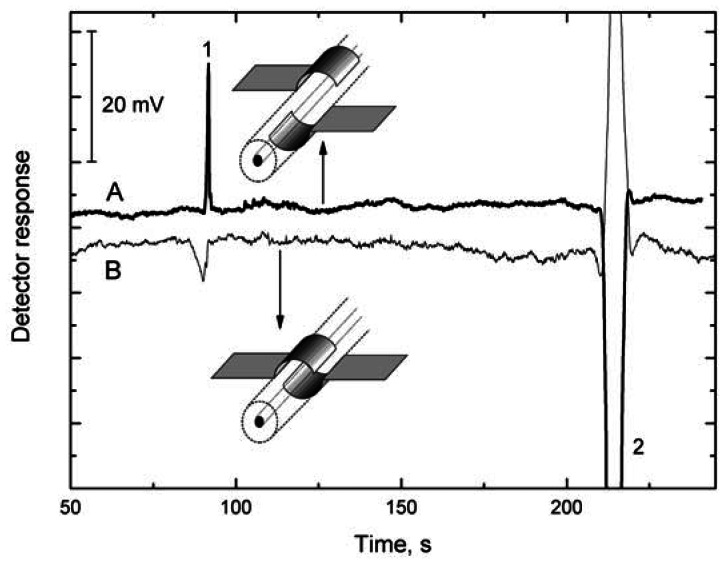
Electropherogram of K^+^ (**1**) ion and water gap (**2**) obtained using the detection cell with semi-tubular electrodes placed opposite one another with a 1 mm gap between them, 50 μM K^+^ (**A**, thick line), and with electrodes completely overlapped, 500 μM K^+^ (**B**). Fused silica capillary, 75 μm i.d., total length/length to detector, 58 cm/50 cm, background electrolyte, 20 mM MES/His, separation voltage/current, 20 kV/11 μA, hydrodynamic sampling, 10 cm/10 s, C^4^D, 300 kH/4.5 V_pp_.

The effect of the width of the gap between the electrodes has been studied [15] on a cell with semitubular electrodes (Figure 2B), one electrode being fixed and the other, opposite, electrode being shifted along the capillary surface. The detection sensitivity, defined as the ratio of the electric current flowing through the cell and specific conductance of solution, *I*/*κ*, increases with decreasing gap width, *d*, between the electrodes [*I*/*κ* is proportional to 1/*d* as follows from Equations (6) and (7)]. The detection sensitivity increases with decreasing *d* from 3 mm up to 0.5 mm. There is a negligible change in the detection sensitivity within the gap width interval from 1 to 0 mm but the sensitivity begins to decrease rapidly when the electrodes gradually become superimposed. This decrease is due to the change in the cell response character—the effect of the capacitance component of solution on the impedance *Z*_1_ increases and that of the resistance component decreases. When the electrodes are placed opposite one another, the cell becomes a virtually pure permittivity detector whose signal is determined predominantly by the change in the test solution permittivity and substantially less by the change in its conductivity. The change in the character of the detector is clearly demonstrated on the electropherogram of the potassium ion and water gap in Figure 5. The response to passage of the potassium ion is high in the conductivity arrangement of the detection cell, because the zone has higher conductivity than conductivity of background electrolyte and the passage of the low-conductivity water gap is registered as a substantial reduction in the response. In the permittivity arrangement of the detection cell, the potassium ion reduces the permittivity of the zone, leading to a reduction in the response, but the response on passage of the water zone is high because the permittivity is higher than permittivity of background electrolyte.

### 4.4. The Effect of the Thickness of the Dielectric Layer

The coupling capacitance *C*_cpl_ in the electric equivalent circuit increases with decreasing thickness of the dielectric; consequently, the capacitance component contributes less to the cell impedance and the current signal increases. The wall thickness of common capillaries for capillary electrophoresis (CE) is approx. 150 μm. When it was decreased by *ca.* 30% by careful filing, then the detection sensitivity was increased by more than 120% in measurements with planar electrodes (Figure 2C) [15]. The filing down is an awkward operation and the capillaries become very fragile; therefore, this is not a potential approach for sensitivity improvement. On the other hand, planar electrodes used in chip electrophoresis can easily be placed very close to the separation channel and the detection sensitivity can be increased substantially [23]. Planar electrodes insulated from the solution by a thin plastic foil have also been used in the thin-layer format of the contactless conductivity detector. The detector was applied to the detection of ionic compounds such as benzoic, lactic and octanesulfonic acids, and sodium capronate, after their separation by high performance liquid chromatography (HPLC) [24]. The properties of the thin-layer detector were compared with those obtained by the standard contactless conductivity detector equipped with tubular electrodes [25]. As compared to the thin-layer cell, the tubular cell was substantially simpler and its fabrication was much easier. In addition, it was found that the tubular cell outperformed the thin-layer cell in the main analytical parameters. A survey of other applications of C^4^D in liquid chromatography is given in reference [24].

A completely different way of obtaining a contactless conductivity cell with a very thin dielectric layer is based on the use of thin insulated wires placed directly in the test liquid stream [26] (see Figure 2D). The lacquer insulation layer has a thickness of *ca.* 1 μm. This approach cannot, of course, be employed with silica capillaries, but it is readily used in classical HPLC or flow-injection analysis (FIA) systems. This cell design has also been used for detection in the gaseous phase [27]. It has been found that the measurement is sufficiently sensitive and reliable for determinations of common concentrations of water in the air. The dynamic characteristics and the signal stability demonstrate that the cell will be suitable for long-term continuous monitoring of the air humidity. Qualitative tests indicate that vapours of organic solvents can also be monitored.

**Figure 6.**
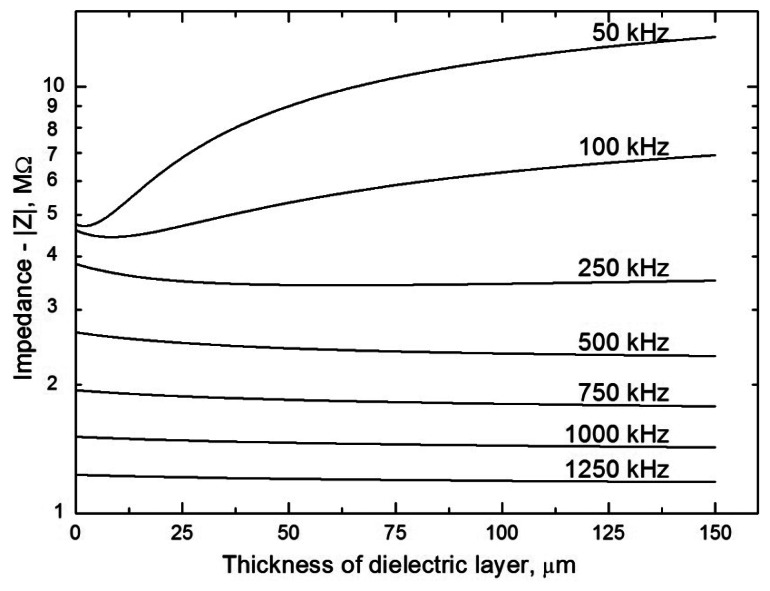
Model dependences of the impedance on the thickness of the dielectric layer in the range from 0.1 to 150 μm for various input voltage frequencies (specified next to the curves, in kHz). Model calculations are based on the equivalent circuit in Figure 1, which was employed for a tubular cell (Figure 2A). The modeling parameters are: silica capillary, inner diameter *r*_1_ = 37.5 μm, thickness of capillary wall 0.1–150 μm, *ε_r_* (fused-silica) = 4.3; *d* = 1 mm, *w* = 2 mm and *C_x_* = 0.1 pF (estimated); the capillary is filled with 20 mM MES/His solution, conductivity *κ* = 4.7 × 10^−2^ S m^−1^.

The effect of the thickness of the dielectric on the impedance of contactless detectors can be modeled, see Figure 6. It follows from the model based on an equivalent circuit applied to a tubular cell (Figure 2A) that the impedance of the sensor increases substantially with the thickness of the dielectric only at low alternating signal frequencies. At high frequencies, the impedance is practically independent of the thickness of the dielectric (with increasing frequency, the resistance of the condenser, *Z*_c_ = −*i*/2*πfC*_cpl_, decreases and the dominant component contributing to the total impedance is then the ohmic resistance of the solution in the capillary, which is not dependent on the frequency). It thus unambiguously follows that impedance measurements can be successfully performed even in thick-walled capillaries assuming that strong capacitive coupling is ensured (high *C*_cpl_), with simultaneous use of a high-frequency alternating signal. It should also be pointed out that the frequency from which the impedance ceases to depend on the thickness of the dielectric also varies substantially with the magnitude of the stray capacitance, *C*_x_.

### 4.5. The Effect of the Input Alternating Voltage Parameters on the Detector Properties

These effects have been studied quite frequently because the investigation is simple experimentally. It should be pointed out that the detection cell performance is also affected by the character and type of the electronic circuitry employed; see, e.g., references [22,28]. In general, the simultaneous effects of all the experimental parameters mentioned in Section 2 should always be kept in mind.

**Figure 7.**
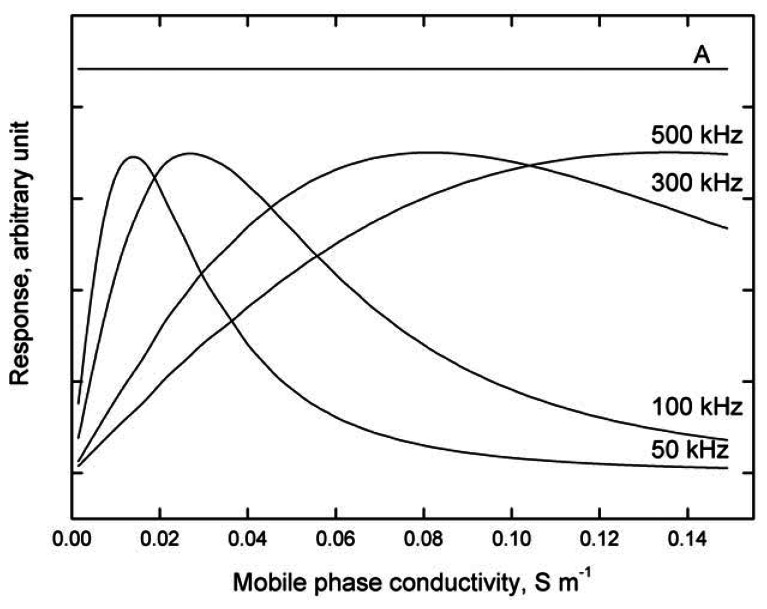
Model dependence of the detector response to a sample with constant conductivity introduced into carrier solutions with various conductivities, at various input voltage frequencies. For comparison, the response produced by cell with purely ohmic resistance is given by straight line A. The modeling parameters are: carrier liquid conductance, *κ_E_*, in a range from 1.5 × 10^−3^ to 1.5 × 10^−1^ S m^−1^ (corresponding to *ca.* 10^−4^ to 10^−2^ M KCl); analyte conductance, and *κ_A_* = 1.5 × 10^−4^ S m^−1^ (corresponding to *ca.* 10^−5^ M KCl); the detected conductance equals *κ_E_* + *κ_A_*, *w* = 2 mm; for the other parameters, see Figure 4.

Figure 7 depicts the results of modeling the detector response dependence, *i.e.*, electric current flowing through the cell, see Equation (6), on addition of a sample with constant conductivity to carrier liquids with various conductivities at various frequencies of the input alternating voltage. It follows that:

a) The optimum frequency, at which the response is largest, increases with increasing conductivity of the test solution; this dependence becomes progressively more pronounced with decreasing conductivity of the test solution.
b) The calibration dependences obtained at constant frequency are non-linear and may even be non-monotonous, especially at lower frequencies.

For analogous modeling results, see reference [29].

**Figure 8.**
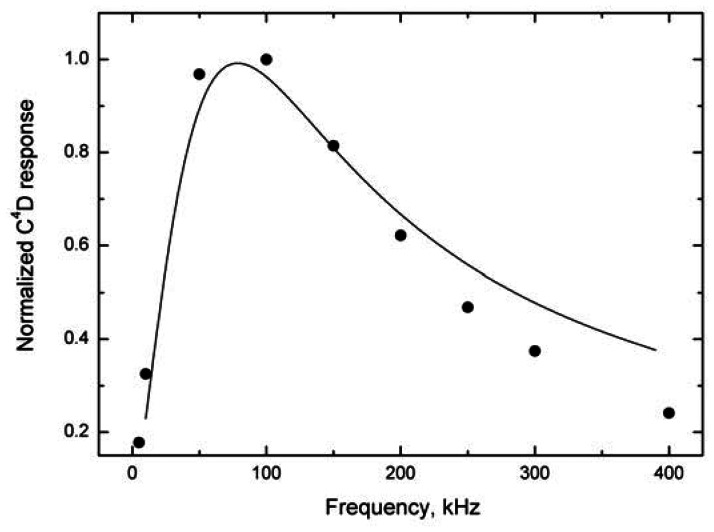
The model (solid line) and experimental (points) dependences of the insulated wire cell response on the input voltage frequency; electrodes placed at right angles to the test liquid flow, Figure 2D (1). The modeling parameters are: *r*_1_ = 4.5 × 10^−5^ m, *r*_2_ = 5 × 10^−5^ m (difference *r*_2_ − *r*_1_ is the dielectric thickness), *w* = 2.54 × 10^−4^ m (the electrode length equals the internal diameter of the PTFE tubing), *d* = 4 × 10^−4^ m, *ε*_r_ = 4 (polyimide insulating film), carrier liquid conductance *κ*_E_ = 7.5 × 10^−5^ S m^−1^ (5 × 10^−6^ M KCl), analyte zone conductance *κ*_A_ = 1.5 × 10^−3^ S m^−1^ (10^−4^ M KCl) and the capacitance used in the model *C*_x_ = 1 pF (estimated). The cell geometry is modeled from the relationship for the board capacitor [Equation (9)].

Our measurements indicate that the model employed for tubular cells is also applicable without substantial limitations to cells with insulated wire electrodes [26] oriented across the tube, see Figure 2D (1). For illustration, the model and experimental response—frequency dependences for a cell with wire electrodes placed at right angles for constant Δ*κ* of the solution are compared in Figure 8. The decrease in the cell response at high frequencies is caused by the increasing influence of the stray capacitance; the operational characteristics of the electronic components used in the detector electronics can also influence the response as mentioned above (the limited bandwidth of operational amplifier). These cells have also been employed for FIA determination of total inorganic carbon in aqueous solutions [30].

The input voltage amplitude has a minor effect. Generally, the greater the amplitude, the higher is the measured signal. The literature records an amplitude range from 0.5 V [19] to 450 V [31], but the most common values are 10 to 20 V, provided by standard AC signal generators. The amplitudes of the input alternating voltage can be an order of magnitude lower if a commercial instrument is used for impedance spectrometry, see references [32,33].

### 4.6. The Cells Designed for Dielectrometric Measurements

The detection cells discussed so far were designed for measurement of the alternating current determined primarily by the resistance component of the cell impedance. Certain cell geometry and properties of the test solution can enhance the cell capacitance component. In the low conductivity solutions for which it holds that *R*_liq_ ≫ (−*i*/*ωC*_liq_), the effect of *R*_liq_ on *Z* can be neglected and the detection cell acts primarily like a condenser. An example is the cell with wire electrodes placed in parallel with the test liquid stream, Figure 2D (2). This cell was connected as an input capacitor in a differentiating circuit, whose output voltage, *U*_0_, is given by:

(9) $$U_{0}=-CR_{f}(dU_{i}/dt)$$

where *C* is the overall capacitance of the cell, *R*_f_ is the feedback resistance of the differentiating circuit and *dU*_i_/*dt* is the change in the input voltage with time. If the input voltage is triangular, *i.e.*, if *dU*_i_/*dt* is constant, and the feedback resistance is also constant, then the output voltage amplitude is proportional to the cell capacitance. This cell design has been tested [34] in detection of mixtures of non-ionic analytes (methanol, dioxane) with water. Lower signal frequencies are involved, of the order of units of kHz, compared to conductance measurements, placing smaller demands on the signal treatment. On the other hand, the detection limit is about one order of magnitude higher than that obtained in conductance measurements with the same cell [26]. The dynamic ranges useful in practice are limited to solution conductances not exceeding *ca.* 0.007 S m^−1^. An advantage over the conductance cells lies in the fact that the concentrations of the non-ionic compounds can be monitored on the basis of the permittivity values; an exponential curve can be constructed through the experimental calibration points and its equation permits determination of the permittivity from the value of the detector response.

Another capacitance cell with two planar electrodes placed opposite each other, Figure 3A, can be connected as a capacitor which determines the frequency of an integrated stable multivibrator (e.g., common type, 4047); the detection cell capacitance can be calculated from the relationship for the board capacitor:

(10) $$C_{\text{liq}}={ɛ}_{0}{ɛ}_{r}S/d$$

where *ɛ*_r_ is the relative permittivity of the test solution, *S* is the electrode area and *d* is the distance between the electrodes. The analytical signal is then represented by the multivibrator frequency. This type of detector has been used for determinations of organic solvents in binary mixtures [35,36]. This approach permits extremely simple, rapid and cheap analyses of binary liquid mixtures; however, it suffers from a serious limitation in that the overall electric conductivity of the mixture must be low, as has already been mentioned above. The pronounced sensitivity of these methods to higher conductivities of test solutions, which is undesirable in determinations of the permittivity, can, on the other hand, be used to detect traces of ionic substances in, e.g., non-aqueous solvents.

The detection cells with tubular electrodes, Figure 3B,C, connected with an astable multivibrator have also been tested for determination of the ethanol content in automotive fuel [37]; the ethanol concentrations determined using the impedance cell agree, within the reliability interval, with those obtained by gas chromatography-mass spectrometry (GC-MS) measurements and fall within the limits recommended by the relevant European Standard.

## 5. Conclusions and Outlook for the Future

Contactless impedance detection has already become a standard part of sensor and detector science. The theoretical background is reasonably well understood, even if modeling and prediction of the cell behavior is satisfactory only for the shapes of dependences and trends, whereas the absolute signal values can be predicted only with difficulties.

The main area of application will definitely remain in the fields of HPLC and CE separations, where C^4^Ds are commercially available [38,39], with emphasis on miniaturized systems, primary in the form of lab-on-the chip designs. Further progress can be expected in the area of instrumental design; an example can be found in reference [40], in which end-to-end differential C^4^D is tested for electrophoresis on a chip. The detection is based on recording the difference in the signals from two identical detectors with planar electrodes, of which one is located at the beginning and the other at the end of the separation channel. This arrangement has the advantage that it increases the signal/noise ratio and minimizes the baseline drift. This leads to a lower detection limit compared to the normal end-column location of the detector. Another promising area lies in the development of new types of combined detectors [41]. A typical arrangement combines C^4^D with optical detection; detectors based on quite different detection principles provide more complex information on the studied system [42–44].

The newest applications demonstrate that the use of impedance detection extends beyond the area of separation techniques. A simple disposable polydimethylsiloxane chip [32] has been developed for contactless impedance flow-through cytometry. In the measurement, the chip is placed on a printed circuit board with electrodes and, as required, the necessary electronics. The equipment enables detection of the passage of individual cells through the chip channel and is intended for the diagnosis of infectious diseases. The development of the chip was motivated by attempts to decrease the cost of individual examinations; a new chip is used for each examination, while the printed circuit board can be used repeatedly. The standard experimental arrangement with cylindrical electrodes (unconventionally made of conductive silicone) located on the outside of a thin-walled glass capillary was used for the detection and measurement of the conductivity of drops of an aqueous salt solution (KCl) generated in a stream of liquid immiscible with water (tetradecane) [45]; the method can be employed in digital microfluidics.

Other measuring techniques in which conductometric electrodes are placed at the outside walls of the detection cell are perspective, e.g., for studying reaction mechanisms in hermetically closed reaction vessels or tubes. The method has already been used to evidence whether the allylation mechanisms of aldehydes proceed via an ionic or neutral pathway [46].
